# Acid–Base, Gas, Ions, and Glucose Analysis in Follicular Fluid in Holstein-Friesian Dairy Cows Is Associated with the Follicle Size in Poland

**DOI:** 10.3390/ani13101636

**Published:** 2023-05-14

**Authors:** Bartosz Pawliński, Monika Petrajtis-Gołobów, Michał Trela, Olga Witkowska-Piłaszewicz

**Affiliations:** Department of Large Animal Diseases with Clinic, Institute of Veterinary Medicine, WULS—SGGW (Warsaw University of Life Sciences, Szkoła Główna Gospodarstwa Wiejskiego), Nowoursynowska 100, 02-797 Warsaw, Poland; bartosz_pawlinski@sggw.edu.pl (B.P.); monika_petrajtis_golobow@sggw.edu.pl (M.P.-G.); michal_trela@sggw.edu.pl (M.T.)

**Keywords:** cattle, oocyte, gasometry, pH, calcium, potassium, fertility, reproduction

## Abstract

**Simple Summary:**

Reproductive failures in dairy farms are extremely costly for breeders; however, the cause of loss is often very hard to determine. The environment may reflect an influence on the quality of the oocyte follicular fluid (FF). It was suggested that FF can be used as an indicator for the functional status of the ovarian follicle in bovine species, however, the topic is still not well known. This study aimed to investigate the variations in FF parameters connected with acid–base balance, ions, and glucose analysis. It was proven that changes in the FF formula were associated with the follicle size.

**Abstract:**

The lack of fertilization and early pregnancy loss is seen in the quality and maturation of the oocytes. The environment of the first divisions and maturation of the oogonia, which is also a reflection of the quality of the oocyte, is the follicular fluid (FF). Thus, the purpose of this study was to investigate the variations in parameters such as pH, pCO_2_, pO_2_, standard HCO_3_^−^, actual HCO_3_^−^, base excess (BE), base excess of extracellular fluid (BE ecf), ctCO_2_, ions: Na^+^, K^+^, actual ionized calcium Ca^2+^, adjusted ionized calcium (at pH 7.4) Ca^2+^ (7.4), Cl^−^, anion gap (AnGap), and glucose in FF collected from different size follicles in dairy cattle. The most apparent differences were related to pH, K^+^, and Ca^2+^ 7.4 concentration in comparison to changes in follicle size (*p* < 0.05). Several trends were also evaluated as an increase in follicular size was followed by an increase in pH, BE, and Ca^2+^ 7.4 value and a decrease in the K^+^ concentration (*p* < 0.05). In conclusion, there are changes in FF formularies associated with the size of follicles. However, more research is necessary to establish the reference value, which then could be one of the factors describing the quality of the follicle and the developmental potential of the associated oocyte.

## 1. Introduction

Poland, with 1.7% of global milk production (14.1 billion liters), is the second largest dairy producer in the European Union [1]. However, the increase in milk production is accompanied by a decrease in the reproductive performance of dairy cows. Decreased reproductive efficiency is mostly connected with estrus cycle disorders. Anestrus is the main cause of ineffective insemination, increased insemination index, reduced fertilization rates, and extended time of inter-pregnancy and inter-calving period, which generates huge economic losses [2]. The above circumstances encourage the search for methods of faster and more effective genetic improvement in dairy cattle breeding and the field corresponding to the emerging needs in reproductive biotechnology. The used biotechniques such as in vivo and in vitro embryo production, are showing a constant increase around the world, even observable during the COVID-19 pandemic [3]. In 2020, more than 1.5 million bovine embryos were recorded, which represents an increase of 7.0% compared with 2019 (1,518,150 vs. 1,419,336, respectively) [3]. However, also in other species, there was a substantial increase in the numbers of in vivo-derived (IVD) (in horses +13.6%; sheep +33.3%; goats +51.0%), as well as in vitro-produced (IVP) embryos (horses +37.1%; goats +204.1%) [3]. Thus, taking into account these numbers, improving the biotechniques used in reproduction is essential. 

However, the biotechnology of reproduction as a very sensitive and developing field requires constant research and a better understanding of the processes such as gamete maturation and fertilization. The most common reproductive biotechnology in all mammalian species is artificial insemination (AI), which includes sperm cryopreservation. In cattle nowadays, embryo production (ET) is the fastest developing segment as mentioned earlier. Thus, in vitro bovine embryo production is important from an economic point of view, as well as in recent years it has become a model for humans. 

Follicular fluid (FF) has been examined for many years to evaluate the oocyte microenvironment. FF provides nourishment for oocyte development such as proteins, lipids, ions, carbohydrates, etc. It strongly influences the environment of oocytes. It was suggested that the main problem of inferior oocyte and embryo quality is the FF quality rather than a disruption in gonadotropin secretion in high-yielding dairy cattle [4,5]. Thus, monitoring of FF composition can be used for non-invasive diagnosis.

The material can be obtained from the ovary of slaughtered animals [4,5,6] as well as from live animals in the course of the ovum during the pick-up procedure [7,8,9]. FF has several oocyte-related functions, creating together with the cumulus cells the intrafollicular environment that supports the acquisition of developmental competence in oocytes. FF also protects the oocyte against proteolysis and extrusion during ovulation [10], thereby influencing in vitro maturation and fertilization competence of bovine oocytes [11]. As a product of the transfer of blood plasma components through the blood-follicular barrier and of the metabolism of theca and granulosa cells, FF contains several hormones such as testosterone, estradiol-17*β* (E2), and progesterone (P4) and some growth factors. In addition, it seems to be convenient that FF might be a perfect maturation medium as it also influences the resumption of meiosis and cytoplasmic maturation of bovine oocytes [4,5]. Thus, optimization of the in vitro maturation environment is vital for generating viable embryos.

As mentioned earlier, diluted FF is also used in media supporting the bovine oocyte maturation in vitro or for the conservation of oocytes. It was postulated that the changes in the composition of FF may be used as an evaluation parameter of oocyte quality, which can be directly related to fertility [12]. In the study performed by Matoba et al., 2014, they confirmed that analysis based on the follicular fluid metabolite profile is the best predictor of oocyte developmental competence [12]. As it was mentioned earlier FF is also used as medium enrichment. Thus, research connected with that topic is of great importance as the FF contains proteins, steroids, glycoproteins, and fatty acids—the compounds which contribute to the metabolism of cells and follicular oocytes. 

However, there are only several studies connected with acid–base balance and gas analysis, and most of them are older than 15 years and/or have only evaluated a limited number of parameters [13,14,15]. Maintenance of the acid–base balance as well as equilibrium in ion concentration is fundamental for the normal functioning of biological processes, mainly due to the pH dependence of enzyme function [16]. Thus, knowledge of changes in those parameters is essential for the oocyte quality understanding. Broadening the knowledge about this topic will help with embryo transfer procedures by obtaining better quality oocytes as well as a more suitable medium for culturing them during maturation. Thus, it may be applied in the future to farm animal production. 

In addition, the embryo production success rate depends also on the follicle size which is probably connected with different FF compositions [10]. In human medicine, the in vitro treatment is follicle size rather than their competence. A good example of that is that the timing of administration of hCG is mostly guided by the size of the lead follicle or lead follicular cohort. However, there is a lack of studies connected with this topic. The hypothesis is that the FF acid–base, gas, and ion composition vary in different size follicles. Thus, this study investigates the changes in the acid–base, gas, and ion concentrations in FF obtained from different size follicles in dairy cows.

## 2. Materials and Methods

### 2.1. Biological Samples

The samples were collected post-mortem in the slaughterhouse, which does not fall under the legislation for the protection of animals used for scientific purposes, national decree-law (Dz. U. 2015 poz. 266), and European Union law (2010-63-EU directive). Thus, no Ethical Committee’s permission was needed for sample retrieval after slaughter.

FF was collected from 44 slaughtered cows (Holstein Friesian—HF breed). The inclusion criteria were good health based on veterinary clinical examination and normal reproductive tracts upon macroscopical examination after slaughter. In addition, the cows were assessed pre-slaughter by ultrasound examination of the reproductive tract. All examined cows were multiparous. Fluid from 1 to 2 follicles was collected from each ovary. The follicles were divided into 3 groups, according to their size: I: 5 to 10 mm diameter (small follicles; *n* = 16); II: 11 to 25 mm (dominant follicles; *n* = 24), III: >30 mm (cysts; *n* = 4). FF was obtained by aspiration directly into 1-mL gasometric and 3-mL plain tubes (Monovette 1mL LH, Sarstedt, Nümbrecht, Germany). The expulsion of the air bubbles from the syringes was performed immediately after collection, syringes were capped with a rubber stopper, and the syringes were stored on ice. FF was centrifuged at 1000× *g* for 1 min and the supernatant was stored at 4 °C until analysis. 

### 2.2. Acid–Base, Gas, and Biochemical Analysis of FF 

The supernatant was examined a maximum of 5 min after slaughtering. The following parameters were marked and calculated: pH; pCO_2_—partial pressure of carbon dioxide; pO_2_—partial pressure of oxygen; HCO_3_-act—actual bicarbonate concentration; HCO_3_-std—standard bicarbonate concentration; BE(B)—base excess; BE ecf—base excess of extracellular fluid; ctCO_2_—total carbon dioxide concentration; Na^+^, K^+^, Ca^2+^—actual ionized calcium; Ca++7.4—adjusted ionized calcium at pH 7.4; Cl^−^, AnGap—anion gap; and cAMP and glucose (Glu) FF concentration. The oximetric parameters were assessed using a critical points analyzer, RAPIDPoint 500 (Siemens, Erlangen, Germany). The biochemical parameters were assessed using the analyzer, BS-120 (Shenzhen, China). 

As there is no recommendation on how to perform the FF evaluation, the measurements were carried out as recommended by the National Committee of Blood Laboratory Standards (Considerations in the Simultaneous Measurement of Blood Gases, Electrolytes and Related Analytes in Whole Blood; Proposed Guidelines). For each test, the analyzer’s operating temperature was set according to the bovine rectal temperature recorded during sampling.

### 2.3. Statistical Analysis

Statistical analysis was performed in PQStat 1.6.4.121. (Poznan, Poland.). The numerical variables were given as the arithmetic median and standard deviation (SD), or the interquartile range (IQR), unless the variable was normally distributed according to the Shapiro–Wilk W test. The range was presented in all cases. Between-group comparisons were performed using the Kruskal–Wallis H test along with Dunn–Bonferroni’s post hoc test, unless the variable was normally distributed. Additionally, a Jonckheere–Terpstra trend test was performed. The significance level was set at *p* < 0.05, and a test probability was considered highly significant when *p* < 0.01.

## 3. Results

The values for the examined FF parameters in cattle from the different groups are presented in Table 1.

**Figure 1:**
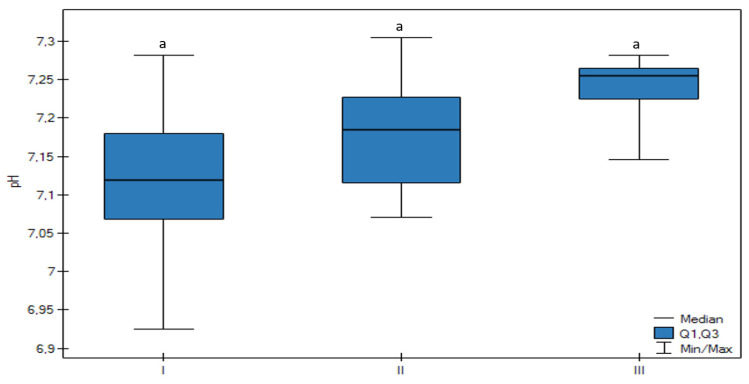
Differences in the pH of the FF in all groups (I, II, III) according to the ovarian follicular size. The upper whisker in the box plots represents the maximum value; the upper quartile is represented by the upper line of the box (Q3), the median is represented by the center line inside the box, the lower quartile is represented by the lower line of the box (Q1), and the minimum value is represented by the lower whisker. Different letters (a) mean statistically significant differences (*p* < 0.05).

**Figure 2:**
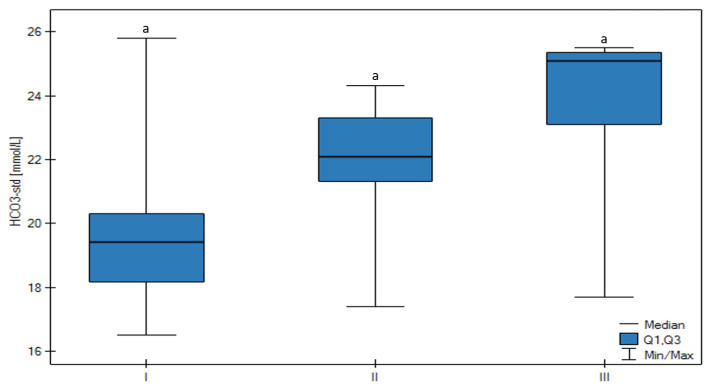
Differences in the HCO_3_ concentration of the FF in all groups (I, II, III) according to the ovarian follicular size. The upper whisker in the box plots represents the maximum value, the upper quartile is represented by the upper line of the box (Q3), the median is represented by the center line inside the box, the lower quartile is represented by the lower line of the box (Q1), and the minimum value is represented by the lower whisker. Different letters (a) mean statistically significant differences (*p* < 0.05).

**Figure 3:**
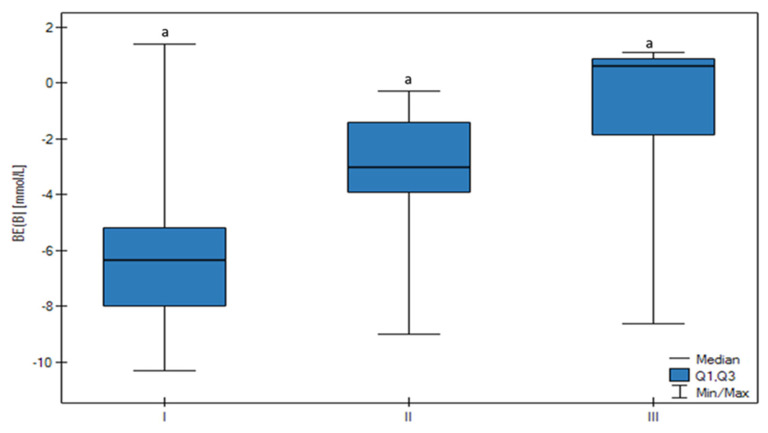
Differences in the BE concentration of the FF in all groups (I, II, III) according to the ovarian follicular size. The upper whisker in the box plots represents the maximum value, the upper quartile is represented by the upper line of the box (Q3), the median is represented by the center line inside the box, the lower quartile is represented by the lower line of the box (Q1), and the minimum value is represented by the lower whisker. Different letters (a) mean statistically significant differences (*p* < 0.05).

The pH was the lowest in Group I in comparison to the other groups (Figure 1). There was no statistically significant difference between groups, but a trend that confirmed that the decrease in follicle size was related to a lower pH value (*p* < 0.05) was observed. There were no significant changes between HCO_3_-std (Figure 2), BE ecf, and BE(B) (Figure 3) values, however, there was a significant trend that confirmed an increase of these parameters connected with the increase in follicle size (*p* < 0.05).

**Figure 4:**
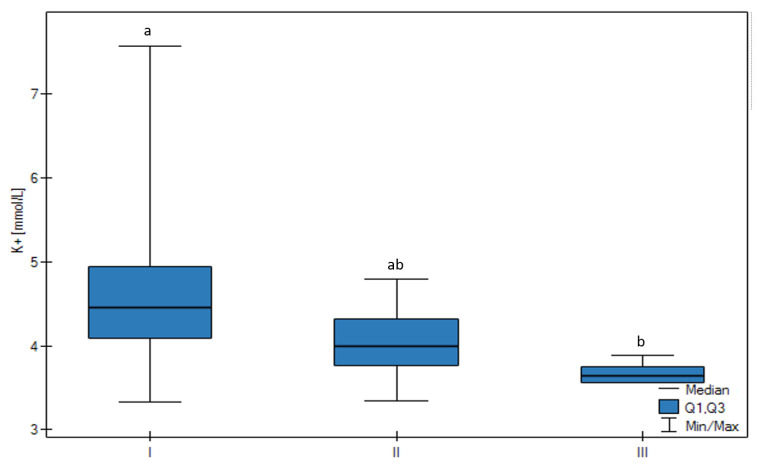
Differences in the K^+^ concentration of the FF in all groups (I, II, III) according to the ovarian follicular size. The upper whisker in the box plots represents the maximum value, the upper quartile is represented by the upper line of the box (Q3), the median is represented by the center line inside the box; the lower quartile is represented by the lower line of the box (Q1), and the minimum value is represented by the lower whisker. Different letters (a, ab, b) mean statistically significant differences (*p* < 0.05).

**Figure 5:**
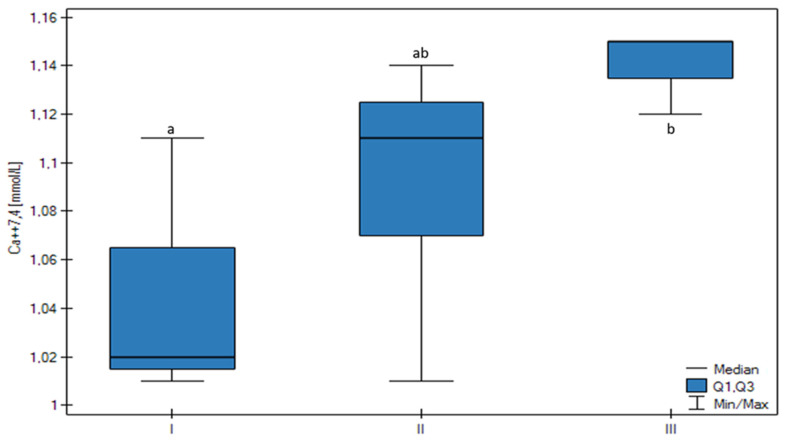
Differences in the Ca2+(7.4) concentration of the FF in all groups (I, II, III) according to the ovarian follicular size. The upper whisker in the box plots represents the maximum value; the upper quartile is represented by the upper line of the box (Q3), the median is represented by the center line inside the box, the lower quartile is represented by the lower line of the box (Q1), and the minimum value is represented by the lower whisker. Different letters (a, ab, b) mean statistically significant differences (*p* < 0.05).

In ions analysis, the concentration of K^+^ and Ca++74 in FF was significantly variable among the groups. The K^+^ concentration was the highest in Group I (*p* < 0.01) and there was a trend confirming that the higher the follicular size, the lower the K^+^ FF concentration (*p* < 0.01) (Figure 4). The Ca++7.4 FF concentration was the lowest in the Group I (*p* < 0.05) and there was a significant positive correlation found between the Ca++7.4 FF concentration and the follicular size (*p* < 0.05) (Figure 5). 

There were no statistically significant changes in pCO_2_, pO_2_ HCO_3_-act ctCO_2_ AnGap, and ions such as Na^+^, Ca^2+^, Cl^−^, and Glu FF concentration between groups.

## 4. Discussion

In the present study, follicular fluid was sampled separately for each cow and each follicle which was unique in comparison to previous studies [13,16]. There are only a limited number of articles published in the changes in acid–base, gas, and ions FF concentrations in comparison to different sized follicles in dairy cattle. Parameters such as pH, BE(B), BE ecf, pCO_2_, pO_2_, HCO_3_-act, HCO_3_-std, AnGap, and ions all together in FF have never been examined in bovine species. Recent studies focus mostly on the follicular fluid metabolome [17,18], whereas routine parameters were never evaluated which may be measured in clinical practice. Of course, pre-ovulatory follicle diameter and follicular fluid metabolome profiles are of high value but because of high cost and low availability, nowadays they cannot be used in veterinary everyday practice.

Acid–base disorders can threaten the proper process of animal reproduction. There are three main fundamental mechanisms: chemical buffering, respiratory regulation, and kidney regulation. There is only one study in which the comparison between values of the routine used acid–base balance parameters in follicular fluid and venous blood in dairy cows and heifers was performed [19]. However, only limited parameters were evaluated such as pH, pCO_2_ and pO_2_, HCO_3_^−^, and BE and there was no evaluation of the influence of follicular size. Thus, the comparison is very hard to perform. 

In our study, the FF pH had a positive correlation with follicular size, which is in line with other findings [9]. It was documented that the tendency for higher pH in FF from follicles derived from stimulated estrus exists. It was suspected that the main cause of that was a change in pCO_2_ in FF, which may be supported by the Henderson–Hasselbach equation [pH = 6.1 + log 10 (HCO_3_^−^/α × pCO_2_)]. However, in our study, the pCO_2_ was constant, whereas the HCO_3_-std was changing as well as BE. Buffer systems carry the most of carbon dioxide (CO_2_), thus pCO_2_ correlates with HCO_3_^−^ values. In many studies, the monitoring of blood pH is suggested as useful for adjusting health, we also found FF pH value as important [20,21].

The HCO_3_^−^ production is related to the ions concentration. Ions such as Na^+^, K^+^, Ca^2+^, and Cl^−^ play major roles in determining the acid–base balance in biological fluids [22]. They have an impact on the acid–base and water balance, the cell membranes pumping systems, and the energy balance influencing the synthesis of milk ingredients such as lactose, etc. [22].

One of the most important ions in reproduction is Ca^2+^ which influences estrogen synthesis. Its concentration increases during follicle development [23], which was also confirmed in our study. In addition, the importance of calcium signaling was confirmed in research connected with various artificial oocyte activation (AOA) methods [24], suggesting a higher fertilization rate when the Ca^2+^ concentration increases. The increased fertilization rate (approximately 29% ICSI vs. approximately 50% ICSI-Ca) was confirmed and it was obtained by ICSI media calcium supplementation (Intracytoplasmic Sperm Injection) compared with the control group in humans [25]. On the other hand, the implantation capacity may be decreased by low calcium concentrations, while impaired development after implantation may be caused by too high a Ca^2+^ level [25]. Increased Ca^2+^ concentration may lead to the atypical expression of calcium-related protein genes or the production of reactive oxygen species (ROS). The dynamics of the Ca^2+^ demand vary significantly from immature to mature oocytes and the cumulus complex. The decrease in Ca^2+^ stores at the end of maturation reflects the activity of Ca^2+^ in the plasma membrane and the decrease in intercellular communication by gap junctions between cumulus cells observed during meiosis. However, the study on low calcium level oocyte in vitro maturation revealed the delayed extrusion of the first polar body (PB1), and the impaired oocyte cytoplasmic maturation, including mitochondrial and endoplasmic reticulum distribution in cattle [26]. This finding suggests that bovine oocytes that are ready for fertilization have a lower requirement for Ca^2+^ stores, but the process of achieving full readiness for fertilization, metaphase II, requires an adequate concentration of calcium. This observation may confirm the difference between bovine and mouse oocytes as previously described by Boni (2002) [4]. However, the exact calcium-dependent mechanism is unknown and studies describing the bovine FF Ca^2+^ concentration are unique. 

In our study, only the Ca++7.4 FF concentration was correlated with the follicular size. This is probably connected with the sample handling. In our study, we also decided to perform Ca++7.4 evaluation to avoid the potential confounding effects of ex vivo changes to serum pH. As a means to correct pH change and avoid the sample handling requirements, laboratorians have developed pH adjustment equations [27,28]. Proteins (particularly albumin) may bind additional hydrogen ions. So, when the pH decreases, hydrogen ions effectively compete with free calcium for available negative charges on proteins, and the protein-bound calcium level decreases whereas the circulating free calcium blood content increases. Thus, measuring the adjusted ionized calcium at pH 7.4 is beneficial for this study.

Furthermore, calcium-activated potassium channels activation is connected with the concentration of Ca^2+^ [29]. Thus, when the K^+^ concentration decreases, the Ca^2+^ concentration increases [30], which is in line with our results. The FF K^+^ concentration was negatively correlated with the size of the follicle. It was documented that the highest FF K^+^ concentration occurs just before ovulation in sheep, mares, and women [23]. This may relate to the permeability of the follicle membrane to K^+^ and Na^+^. It was postulated that during the activation of murine oocytes, the membrane potential changes [31]. The decrease in K^+^ concentration is associated with the development of the follicle which was also confirmed in our study. K^+^ ions are transferred from the extracellular to the intracellular space. There are a limited number of such studies performed on cattle. A recent study showed that the average concentrations of sodium and potassium in FF in heifers (Na: 139.87 ± 1.83 mEq/L; K: 3.96 ± 0.12 mEq/L) were lower than in cows in lactation (Na: 138.60 ± 1.75 mEq/L; K: 4.00 ± 2.06 mEq/L), however, were still very similar to our results [31]. 

Other ions also participate in oocyte activation, such as Na^+^ and Cl^−^. In our study, the Na^+^ FF concentration was not dependent on the follicular size as well as Cl^−^. It was documented that Cl^−^ ions activate the steroidogenesis connected with luteinizing hormone stimulation in chicken granular cells and the adrenal glands of rats [29]. In cattle, the Cl^−^ FF concentration in lactating cows was higher (120.80 ± 3.40 mEq/L) than in heifers (97.90 ± 3.73 mEq/L) [30] which was probably connected with the decreased steroidogenesis in lactating cows compared to dairy heifers during the estrus. The Na^+^, Cl^−^, and K^+^ concentrations in the FF were similar to those given in other studies [13,14,19,32,33].

In one study, the glucose level decreased with follicle size, however, the distinction between follicles was different than in our study (small < 4, medium = 6–8, large > 10 [mm]) [9]. The authors summarized that glucose metabolism is less intensive in large follicles compared with small ones or it relates to an increasing amount of follicular fluid. However, a more recent metabolomic study confirmed that altered glucose likely contributes to reduced developmental competence of oocytes [17]. Thus, the glucose concentration should be constant, which is in line with our findings as well as others [33,34]. 

The authors are aware that a higher number of cows would be beneficial for the strength of obtained results. Thus, the main limitation of our study is limited number of animals. Due to that, we were unable to establish reference values for gas and ion concentrations in FF. The American Society for Veterinary Clinical Pathology guidelines are very strict in determining the reference values in animals [34]. Thus, further analysis connected with the larger dataset would be beneficial in defining acid–base, gas, and ion FF ranges in dairy cattle.

In addition, the preanalytical changes have been reported during the acid–base and gas analysis. However, the sampling procedure was carried out anaerobically and the special glass syringes dedicated to this purpose were used. In addition, the analysis was performed as fast as possible in the slaughterhouse. Thus, we have made every effort to minimize the preanalytical changes in FF parameter values. In addition, we are aware that methodology or laboratory equipment may influence the differences obtained for the parameters. However, we evaluated the measurements in the routinely used equipment. Nutrition may also influence the ion concentration which is called the dietary cation–anion difference (DCAD) [35,36]. However, it was documented that most of the ions in FF are a result of local metabolism, not connected with blood concentration [9,19]. 

## 5. Conclusions

In conclusion, our study proved there are differences in pH and K^+^ and Ca^++^ FF concentration an association with the size of follicles. These findings may expand knowledge connected with oocyte health evaluation as well as medium composition for in vitro fertilization. Electrolyte as well as acid–base disturbances are a threat to the proper animal reproduction process. However, still, in cattle, there are a lack of studies related to abnormalities in oocyte health which is crucial to prevent the emergence of further breeding problems more effectively.

## Figures and Tables

**Table 1:** Selected acid–base, gas, ions, and glucose concentrations were measured in follicular fluid in three groups depending on the size of the ovarian follicle. Data are presented as arithmetic mean and standard deviation. Different letters (a, ab, b) mean statistically significant difference (*p* < 0.05). Data with ^ present a significant trend. Group I: 5 to 10 mm diameter (small follicles; *n* = 16); II: 11 to 25 mm (dominant follicles; *n* = 24), III: >30 mm (cysts; *n* = 4).

	Group I		Group II		Group III	
Parameters	Arithmetic Mean	Standard Deviation	Arithmetic Mean	Standard Deviation	Arithmetic Mean	Standard Deviation
pH	7.12 ^	0.10	7.18 ^	0.07	7.23 ^	0.06
pCO_2_ [mmhg]	85.81	28.34	77.35	18.72	66.20	3.41
pO_2_ [mmhg]	174.83	26.16	170.84	22.47	171.60	25.10
HCO_3_-act [mmol/L]	26.44	4.84	28.08	4.29	27.63	4.24
HCO_3_-std [mmol/L]	19.85 ^	2.80	21.58 ^	2.24	23.35 ^	3.77
BE ecf [mmol/L]	−2.90	4.89	−0.23	4.32	0.13	5.23
BE(b) [mmol/L]	−5.84 ^	3.50	−3.62 ^	2.84	−1.58 ^	4.69
ctCO_2_ [mmol/L]	29.08	5.47	30.45	4.75	29.70	4.36
Na^+^ [mmol/L]	140.18	3.79	141.95	3.67	140.65	0.44
K^+^ [mmol/L]	4.71 ^a^	1.07	4.04 ^ab^	0.39	3.68 ^a^	0.16
Ca^++^ [mmol/L]	1.17	0.06	1.17	0.05	1.19	0.05
Ca++7.4 [mmol/L]	1.05 ^a^	0.06	1.10 ^ab^	0.04	1.14 ^b^	0.02
Cl^−^ [mmol/L]	103.44	2.71	105.08	3.84	102.75	1.50
AnGap [mmol/L]	15.03	5.30	12.83	3.31	13.93	3.19
Glu [mg/dL]	90.81	30.95	99.42	26.89	90.50	31.82
cAMP [pmol/mL]	2.91	2.45	5.20	5.08	---	---

## Data Availability

The data presented in this study are available on request from the corresponding author.

## References

[1] The Embassy of the Netherlands in Poland Quick Scan Polish Dairy Sector Contents. https://www.agroberichtenbuitenland.nl/actueel/nieuws/2020/10/21/quick-scan-polish-dairy-sector.

[2] Senger P.L. (1994). The Estrus Detection Problem: New Concepts, Technologies, and Possibilities. J. Dairy Sci..

[3] Joao H.V. (2021). 2020 Statistics of embryo production and transfer in domestic farm animals. Embryo Technol. Newsl..

[4] Edwards R.G. (1974). Developmental Potential in Bovine Oocytes Is Related to Cumulus-Oocyte Complex Grade, Calcium Current Activity, and Calcium Stores. Reproduction.

[5] Leroy J.L.M.R., Vanholder T., Delanghe J.R., Opsomer G., Van Soom A., Bols P.E.J., de Kruif A. (2004). Metabolite and ionic composition of follicular fluid from different-sized follicles and their relationship to serum concentrations in dairy cows. Anim. Reprod. Sci..

[6] Orsi N.M., Gopichandran N., Leese H.J., Picton H.M., Harris S.E. (2005). Fluctuations in bovine ovarian follicular fluid composition throughout the oestrous cycle. Reproduction.

[7] Ginther O.J., Kot K., Kulick L.J., Wiltbank M.C. (1997). Sampling follicular fluid without altering follicular status in cattle: Oestradiol concentrations early in a follicular wave. Reproduction.

[8] Jorritsma R., Groot M.W.d., Vos P.L.A.M., Kruip T.A.M., Wensing T., Noordhuizen J.P.T.M. (2003). Acute fasting in heifers as a model for assessing the relationship between plasma and follicular fluid NEFA concentrations. Theriogenology.

[9] Leroy J.L.M., Vanholder T., Delanghe J., Opsomer G., Van Soom A., Bols P.E., Dewulf J., de Kruif A. (2004). Metabolic changes in follicular fluid of the dominant follicle in high-yielding dairy cows early post partum. Theriogenology.

[10] Guerreiro T.M., Gonçalves R.F., Melo C.F.O.R., de Oliveira D.N., Lima E.d.O., Visintin J.A., de Achilles M.A., Catharino R.R. (2018). A Metabolomic Overview of Follicular Fluid in Cows. Front. Vet. Sci..

[11] Covelo I., Puente M.A., Tartaglione C.M. (2022). Influence of Follicular Fluid on in Vitro Maturation and Fertilization of Bovine Oocytes. Open J. Anim. Sci..

[12] Matoba S., Bender K., Fahey A.G., Mamo S., Brennan L., Lonergan P., Fair T. (2014). Predictive value of bovine follicular components as markers of oocyte developmental potential. Reprod. Fertil. Dev..

[13] Berg M.C., Beaumont S.E., Peterson A.J., Berg D.K. (2004). 335 A Procedure Combining ISTAT^®^ Analysis with OPU to Study Bovine Follicular Environments. Reprod. Fertil. Dev..

[14] Cech S., Dolezel R., Lopatarova M., Pechova A. (2007). Acid-base balance of follicular fluid in dairy heifers. Reprod. Domest. Anim..

[15] Quade B.N., Parker M.D., Occhipinti R. (2021). The therapeutic importance of acid-base balance. Biochem. Pharmacol..

[16] Hammon D.S., Wang S., Holyoak G.R. (2000). Ammonia concentration in bovine follicular fluid and its effect during in vitro maturation on subsequent embryo development. Anim. Reprod. Sci..

[17] Read C.C., Edwards L., Schrick N., Rhinehart J.D., Payton R.R., Campagna S.R., Castro H.F., Klabnik J.L., Horn E.J., Moorey S.E. (2021). Correlation between Pre-Ovulatory Follicle Diameter and Follicular Fluid Metabolome Profiles in Lactating Beef Cows. Metabolites.

[18] Wang Z., Song Y., Sun S., Zhao C., Fu S., Xia C., Bai Y. (2022). Metabolite Comparison between Serum and Follicular Fluid of Dairy Cows with Inactive Ovaries Postpartum. Animals.

[19] Indrova E., Dolezel R., Novakova-Mala J., Pechova A., Zavadilova M., Cech S. (2017). Impact of acute metabolic acidosis on the acid-base balance in follicular fluid and blood in dairy cattle. Theriogenology.

[20] Sayers R.G., Kennedy A., Krump L., Sayers G.P., Kennedy E. (2016). An observational study using blood gas analysis to assess neonatal calf diarrhea and subsequent recovery with a European Commission-compliant oral electrolyte solution. J. Dairy Sci..

[21] Edwards R.G. (1974). Arterial blood gases made easy. Reproduction.

[22] Zebeli Q., Mansmann D., Steingass H., Ametaj B.N. (2010). Balancing diets for physically effective fibre and ruminally degradable starch: A key to lower the risk of sub-acute rumen acidosis and improve productivity of dairy cattle. Livest. Sci..

[23] Hassan M.S., Al-Nuaimi A.J., Al-Yasari A.M., Jameel Y.J. (2018). Study the Effects of Follicular Size on some Biochemical Follicular Fluid Composition in She Camel (*Camelus dromedarius*). Adv. Anim. Vet. Sci..

[24] Sun B., Yeh J. (2021). Calcium Oscillatory Patterns and Oocyte Activation During Fertilization: A Possible Mechanism for Total Fertilization Failure (TFF) in Human In Vitro Fertilization?. Reprod. Sci..

[25] Popkiss S., Horta F., Vollenhoven B., Green M.P., Zander-Fox D. (2022). Calcium chloride dihydrate supplementation at ICSI improves fertilization and pregnancy rates in patients with previous low fertilization: A retrospective paired treatment cycle study. J. Assist. Reprod. Genet..

[26] Meng L., Hu H., Liu Z., Zhang L., Zhuan Q., Li X., Fu X., Zhu S., Hou Y. (2021). The Role of Ca^2+^ in Maturation and Reprogramming of Bovine Oocytes: A System Study of Low-Calcium Model. Front. Cell Dev. Biol..

[27] Kur D.K., Hillig T., Hansen S.I., Goharian T., Witte M.L., Thode J. (2020). Evaluation of a New Automated Routine Measurement for Serum Adjusted Ionized Calcium (at pH 7.4) in Patients Suspected of Calcium Metabolic Disease. J. Appl. Lab. Med.

[28] Lam V., Dhaliwal S.S., Mamo J.C. (2013). Adjustment of ionized calcium concentration for serum pH is not a valid marker of calcium homeostasis: Implications for identifying individuals at risk of calcium metabolic disorders. Ann. Clin. Biochem..

[29] Kim J.-M., Song K.-S., Xu B., Wang T. (2020). Role of potassium channels in female reproductive system. Obstet. Gynecol. Sci..

[30] Gałęska E., Wrzecińska M., Kowalczyk A., Araujo J.P. (2022). Reproductive Consequences of Electrolyte Disturbances in Domestic Animals. Biology.

[31] Saleh A., Abozed G.F., Zanouny A.I. (2020). Effect of Different Dietary Electrolyte Balance Levels on Physiological Responses and Metabolic Rate of Rams Exposed to Heat Stress Conditions. J. Anim. Poult. Prod..

[32] Mogheiseh A., Kafi M., Golestani N., Roshan-Ghasrodashti A., Nazifi S., Mirzaei A. (2019). Follicular fluid composition of ovulatory follicles in repeat breeder Holstein dairy cows. Asian Pacif. J. Reprod..

[33] Hussein H., Boryczko Z., Bostedt H. (2013). Acid-Base Parameters and Steroid Concentrations in Pre-Ovulatory Follicles and Plasma of Lactating Dairy Cows with Spontaneous and Synchronized Oestrus or Follicular Cyst. Reprod. Domest. Anim..

[34] Abd Ellah M.R., Hussein H.A., Derar D.R. (2010). Ovarian follicular fluid constituents in relation to stage of estrus cycle and size of the follicle in buffalo. Vet. World.

[35] Friedrichs K.R., Harr K.E., Freeman K.P., Szladovits B., Walton R.M., Barnhart K.F., Blanco-Chavez J. (2012). ASVCP reference interval guidelines: Determination of de novo reference intervals in veterinary species and other related topics. Vet. Clin. Pathol..

[36] Gianesella M., Morgante M., Cannizzo C., Stefani A., Dalvit P., Messina V., Giudice E. (2010). Subacute Ruminal Acidosis and Evaluation of Blood Gas Analysis in Dairy Cow. Vet. Med. Int..

